# NCF-1 plays a pivotal role in the survival of adenocarcinoma cells of pancreatic and gastric origins

**DOI:** 10.1007/s11626-024-00994-0

**Published:** 2024-12-12

**Authors:** Chiemi Furuya-Ikude, Akane Kitta, Naoko Tomonobu, Yoshihiro Kawasaki, Masakiyo Sakaguchi, Eisaku Kondo

**Affiliations:** 1 Division of Tumor Pathology, NIR-PIT Research Institute, Kansai Medical University, 2-5-1 Shinmachi, Hirakata, Osaka 573-1010 Japan; 2 Department of Cell Biology, Okayama University Graduate School of Medicine, Dentistry, and Pharmaceutical Sciences, 2-5-1 Shikata-Cho, Kita-Ku, Okayama, 700-8558 Japan

**Keywords:** NCF-1 (p47phox), ROS, Cancer, Tumor growth, Apoptosis

## Abstract

Reactive oxygen species (ROS) play a pivotal biological role in cells, with ROS function differing depending on cellular conditions and the extracellular environment. Notably, ROS act as cytotoxic factors to eliminate infectious pathogens or promote cell death under cellular stress, while also facilitating cell growth (via ROS-sensing pathways) by modifying gene expression. Among ROS-related genes, *neutrophil cytosolic factor-1* (*NCF-1*; *p47phox*) was identified as a ROS generator in neutrophils. This product is a subunit of a cytosolic NADPH oxidase complex activated in response to pathogens such as bacteria and viruses. NCF-1 has been examined primarily in terms of ROS-production pathways in macrophages and neutrophils; however, the expression of this protein and its biological role in cancer cells remain unclear. Here, we report expression of NCF-1 in pancreatic and gastric cancers, and demonstrate its biological significance in these tumor cells. Abundant expression of NCF-1 was observed in pancreatic adenocarcinoma (PDAC) lines and in patient tissues, as well as in gastric adenocarcinomas. Accumulation of the protein was also detected in the invasive/metastatic foci of these tumors. Unexpectedly, BxPC-3 underwent apoptotic cell death when transfected with a small interfering RNA (siRNA) specific to *NCF-1*, whereas the cells treated with a control siRNA proliferated in a time-dependent manner. A similar phenomenon was observed in HSC-58, a poorly differentiated gastric adenocarcinoma line. Consequently, the tumor cells highly expressing NCF-1 obtained coincident accumulation of ROS and reduced glutathione (GSH) with expression of glutathione peroxidase 4 (GPX4), a quencher involved in ferroptosis. Unlike the conventional role of ROS as a representative cytotoxic factor, these findings suggest that NCF-1-mediated ROS generation may be required for expansive growth of PDAC and gastric cancers.

**Supplementary Information:**

The online version contains supplementary material available at 10.1007/s11626-024-00994-0.

## Introduction

Tumor cells attain growth and progression by regulating the intracellular environment through specific and complicated gene signaling as well as by altering and responding to the extracellular tumor microenvironment. The resulting changes in genetic regulation create certain metabolic alterations in tumor cells, one of which consists of the induction of reactive species (ROS), namely, redox regulation by proteins encoded by oxidase-related genes. ROS have been reported to have conflicting functions depending on various cellular conditions, notably affecting the regulation of cell survival and death (Trachootham *et al.* 2010; Acharya *et al.* 2015). Moderate levels of ROS are known to promote the proliferation and survival of tumor cells, while excess amounts of ROS are known to cause cell damage and death. NADPH oxidase is a multiprotein enzyme complex that serves as the primary generator of intracellular ROS, and neutrophil cytosolic factor-1, the protein encoded by *p47phox* (*NCF1*), is a key regulatory component of this complex (Meijles *et al.* 2014; Tlili *et al.* 2023). Previous reports have studied the expression and biological role of NCF-1 in relation to NADPH oxidase activation in neutrophils, primarily in the context of inflammation (Nagarkoti *et al.,* 2018; Belambri *et al.* 2022). However, the expression and biological roles of NCF-1 in malignant tumors remain poorly defined. Here, we report that NCF-1 is expressed not only in inflammatory cells but also in pancreatic and gastric cancer tissues, as well as in related tumor cell lines, observations suggesting that NCF-1 plays a biological role in producing ROS and contributing to tumor cell survival.

## Materials and methods

### Tissues and cell culture

Formalin-fixed, paraffin-embedded (FFPE) surgical specimens derived from patients diagnosed with gastric cancer (totally 15 cases; tubular adenocarcinoma, well differentiated (diff.) type (tub1) 8 cases, moderately diff. type (tub2) 4 cases, poorly diff. adenocarcinoma (por) 3 cases) and pancreatic adenocarcinoma (totally 10 cases; invasive ductal adenocarcinoma, well diff, type 5 cases, moderately diff. type 4 cases, mucinous type 1 cases) were obtained from the Department of Anatomic Pathology at Hirosaki University. The use of these samples was approved by the ethics committees at both Kansai Medical University and Hirosaki University. The following cell lines were used in this study: HSC-58 (Research Resource Identifier (RRID): CVCL_A391; human gastric signet ring cell adenocarcinoma), obtained from the Japanese Collection of Research Bioresources (JCRB, Ibaraki, Japan) Cell Bank as JCRB1881; BxPC-3 (RRID: CVCL_0186, American Type Culture Collection (ATCC, Manassas, VA); CRL-1687; human pancreatic adenocarcinoma), obtained from ATCC; and HeLa (RRID: CVCL_0030; human papillomavirus-related cervical adenocarcinoma), obtained from the RIKEN BioResource Research Center (BRC; Tsukuba, Japan) as RCB0007. The cells were grown at 37°C in a 5% CO_2_ incubator in RPMI 1640 medium (Wako FUJIFILM Catalog #189–02,025, Tokyo, Japan) supplemented with 10% fetal bovine serum. The identities of the human cell lines were authenticated using short tandem repeat (STR) profiling within the 6 mo preceding use in the present study (supplementary data; cell line authentication). All experiments were performed with mycoplasma-free cells, as confirmed using a mycoplasma detection polymerase chain reaction (PCR) assay following the manufacturer’s instruction (Fig. S1) (PCR Mycoplasma Detection Set, TaKaRa Catalog #6601, Shiga, Japan).

### Immunohistochemistry, immunofluorescence, and immunoblotting

FFPE specimens derived from patient tissues and cell lines were analyzed for production of the proteins of interest using immunohistochemistry (IHC) and immunofluorescence (IF). The primary antibodies used for these analyses included the following: mouse anti-p47[phox] antibody (NCF-1, monoclonal; 1:200; Becton Dickinson, Tokyo, Japan), rabbit anti-glutathione synthetase antibody (GSS, polyclonal; 1:500, Proteintech, Tokyo, Japan), and rabbit anti-mouse glutathione peroxidase 4 antibody (GPX4, monoclonal; 1:1000, Proteintech, Tokyo). For conventional IHC, horse radish peroxidase (HRP)–conjugated goat anti-mouse and anti-rabbit immunoglobulin cocktails (undiluted; Nichirei Biosciences, Tokyo, Japan) were used as the secondary antibodies. For IF, Alexa-488-labeled or Alexa-555 goat anti-mouse or Alexa-555-labeled rabbit immunoglobulin G (IgG; 1:300, Thermo Fisher Scientific, Waltham, MA) secondary antibodies were used according to the species used for the primary antibody. FFPE specimens were subjected to heat-induced antigen retrieval, then treated with absolute methanol containing 0.3% H_2_O_2_ (20 min at room temperature) to block endogenous peroxidases prior to incubation with the primary antibody (12 h at 4°C) for IHC. For IF, the step of blocking endogenous peroxidases was omitted. All immunoblot analyses were performed using cell lysates prepared using the sodium dodecyl sulfate (SDS) sample buffer under standard conditions for each of the specific antibodies mentioned above. For the secondary antibody, HRP-conjugated goat anti-mouse IgG or goat anti-rabbit IgG was used; the secondary antibodies were diluted 10,000-fold with phosphate-buffered saline (PBS).

### In vitro ROS and reduced glutathione detection assay

Intracellular ROS production was detected using a cell-permeable reagent that emits fluorescence at an emission maximum of ~ 488 nm upon oxidation by a ROS (CellROX Green Reagent; Invitrogen, Thermo Fisher Scientific, Waltham, MA). The cells were incubated with the reagent for 60 min at 37°C in a CO_2_ incubator. For combination of immunofluorescence after ROS detection, the cells were further fixed with 10% buffered formalin and subsequently immunofluorescence staining was performed. For detection of reduced glutathione, we used an intracellular dye that reacts with reduced thiols, namely, reduced glutathione, inside viable cells (ThiolTracker Violet; Invitrogen/Thermo Fisher Scientific). This dye exhibits fluorescence excitation/emission maxima of 404/526 nm, respectively. As was the same in the case detecting endogenous protein expression after treatment with ROS tracer, the cells were fixed with 10% buffered formalin; then, subsequent immunofluorescence was performed.

### RNA interference and cell proliferation assay

A predesigned small interfering RNA (siRNA) for human *NCF-1* (ON-TARGETplus, ID L-180696–01–0010) and a control siRNA (ID D0018101005) were purchased from Dharmacon (Horizon Discovery, Cambridge, UK). Cells were transfected with siRNAs (final concentration 20 nM) using the Lipofectamine RNAiMAX reagent (Thermo Fisher Scientific). Forty-eight to 96 h after transfection of the siRNA, the cells were subjected to morphological, immunoblotting, and cell proliferation assays (PremixWST-1 Cell Proliferation Assay System, Takara-Bio, Shiga, Japan).

## Results

### Expression of NCF-1 in tumor tissues

**Figure 1.**
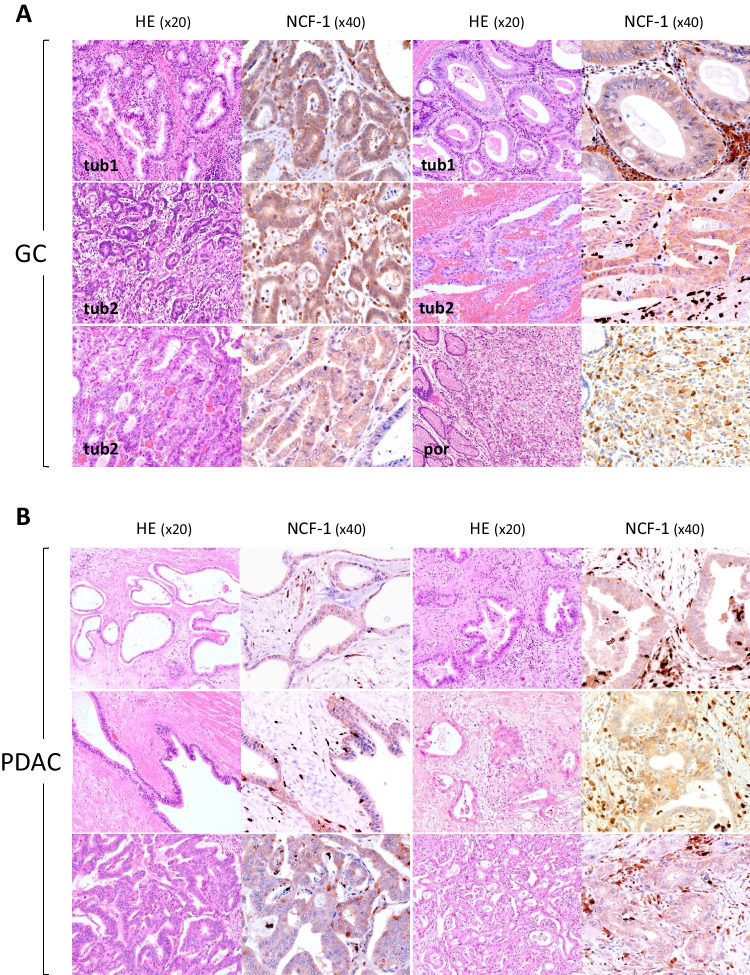
Expression of neutrophil cytosolic factor-1 (NCF-1) in patient tumor tissues. (*A*) NCF-1 expression in representative gastric adenocarcinoma tissues (two cases of tubular adenocarcinoma well diff. type (tub1), three cases of tubular adenocarcinoma moderately diff. type (tub2), and one case of poorly diff. adenocarcinoma (por) of gastric cancer; GC). HE, hematoxylin–eosin staining (× 20); NCF-1, immunohistochemistry using anti-NCF-1 antibody (× 40). (*B*) NCF-1 expression in six cases of patient pancreatic invasive ductal adenocarcinoma (PDAC) tissues (*upper* and *middle panels*, four cases of PDAC well diff. type; *lower panel*, two cases of PDAC moderately diff. type). All cases revealed NCF-1-positive. HE, hematoxylin–eosin staining (× 20); NCF-1, immunohistochemistry using anti-NCF-1 antibody (× 40).

**Figure 2.**
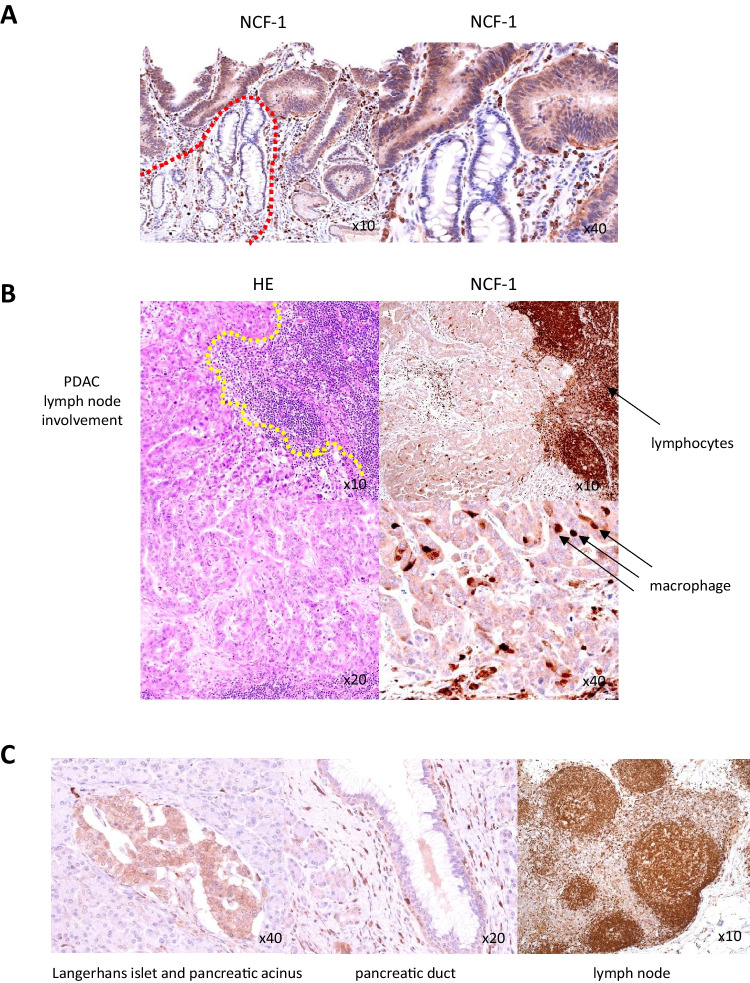
Difference in expression of NCF-1 between tumor cells and normal components. (*A*) Tumor gland distinctly expressed NCF-1 in contrast to normal foveolar of the stomach (left; × 10, right; × 40). *Hatched line* (*red*) shows border between tumor and non-neoplastic areas. (*B*) Metastatic focus of PDAC retains NCF-1 expression. Border of metastatic tumor area is shown with *hatched line* (*yellow*) (*upper panel*; HE × 10, NCF-1 × 10, *lower panel*; HE × 20, NCF-1 × 40). *Arrows* indicate tumor tissue–intermingled inflammatory cells such as lymphocytes and macrophages. (*C*) NCF-1 expression in normal components of PDAC tissue. *Left*, Langerhans islet was positive for NCF-1, while pancreatic acinus was negative (× 40). *Middle*, pancreatic duct was very weak positive for NCF-1 (× 20). *Right*, lymphoid cells, especially germinal center cells, were strongly positive for NCF-1 (× 10).

First, endogenous expression of NCF-1 was examined in patient cancer tissues. IHC employing an anti-NCF-1 antibody revealed staining for endogenous NCF-1 in the cytoplasm of gastric adenocarcinoma cells as well as in inflammatory cells, including neutrophils, lymphocytes, and macrophages (Fig. 1*A*, six representative positive cases). Expression of NCF-1 was detected in 14 cases of patient gastric cancer tissues and it seemed not depending on histologic subtypes, although only one out of the total 15 cases (case of por.) was negative. Intensity of NCF-1 expression in PDAC tissues was comparably weaker than that in gastric cancer tissues, but cytoplasmic staining for NCF-1 was also observed in all 10 cases of invasive pancreatic ductal adenocarcinomas (PDACs) in both tumor nests and inflammatory cells (Fig. 1*B*, six representative cases). Normal foveolar epithelia displayed no or very weak staining for NCF-1, which made clear difference in expression level of NCF-1 on cancer cells (Fig. 2*A*). Metastatic foci of PDACs to lymph nodes retained NCF-1 expression on the cancer cells, which was similar to that seen at the primary sites (Fig. 2*B*). As a non-neoplastic component, strong expression of NCF-1 was seen at Langerhans islet, while pancreatic acinar cells barely expressed it (Fig. 2*C*, left). Pancreatic ductal cells also barely expressed NCF-1, which are recognized to be an origin of PDAC (Fig. 2*C*, middle). On the contrary, robust expression of NCF-1 was observed in inflammatory cells intermingled in PDAC tissues including lymphocytes, neutrophils, and macrophages (Fig. 2*B* and *C*). Especially, germinal centers of the lymph node, in where apoptosis is strongly activated, highly expressed NCF-1 (Fig. 2*C*, right).

### Biological role of NCF-1 in *cancer* cells

**Figure 3.**
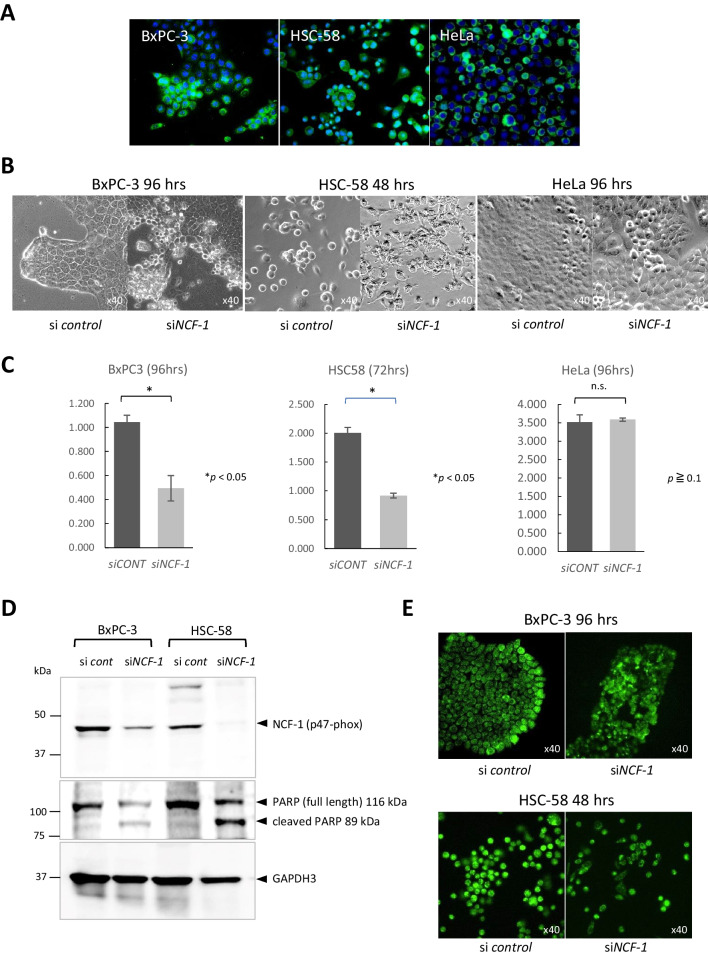
Effect of RNA interference with siRNA specific to the gene encoding neutrophil cytosolic factor-1 (NCF-1). (*A*) Expression of NCF-1 in tumor lines BxPC-3, HSC-58, and HeLa detected by immunofluorescence (× 20). (*B*) Induction of cell death (as assessed by morphology) in BxPC-3 cells (96 h), HSC-58 cells (48 h), and HeLa cells (96 h) at the indicated times after transfection with si*NCF-1*. Unlike BxPC-3 and HSC-58, HeLa did not show significant cellular damage. (*C*) Cell proliferation assay of BxPC-3, HSC-58, and HeLa following transfection with si*NCF-1*. For both BxPC-3 and HSC-58, the results showed statistical significance (*p* < 0.05) between cultures transfected with control and *NCF-1* small interfering RNAs (siRNAs). Vertical axis: measured value (absorbance A450–A690 nm). Means and s.d. of triplicates are shown. (*D*) Immunoblotting of si*NCF-1*-transfected BxPC-3 and HSC-58 cell lysates using anti-poly-ADP ribose polymerase (PARP) antibody, demonstrating an increase of cleaved PARP (a marker of apoptosis) to lysate from the control siRNA-transfected cells, respectively. (*E*) Reactive oxygen species (ROS) generation in cells subjected to transfection with control or *NCF-1* siRNAs. BxPC-3 and HSC-58 are shown at 96 and 48 h post-transfection (respectively; × 40).

Based on the results obtained with patient tumor tissues, three tumor cell lines were selected for further analysis. By immunofluorescence using anti-NCF-1 antibody, BxPC-3 (as a representative PDAC line), HSC-58 (as a representative gastric adenocarcinoma line), and HeLa (as a representative uterine cervical squamous cell carcinoma line) were robustly expressing NCF-1 (Fig. 3*A*).

To address the role of NCF-1 expression in cancer cells, we employed an RNA interference assay using siRNA specific to *NCF-1* (si*NCF-1*) on BxPC-3, HSC-58, and HeLa. The exposure of BxPC-3 cells to si*NCF-1* triggered prominent cell death at 72 to 96 h post-transfection, as evidenced by cell morphology. Notably, this effect was started to be observed at growth front (marginal zone) of the tumor nest, which eventually involved in whole tumor nests (Fig. 3*B*, left panel). Morphological analysis of HSC-58 cells transduced si*NCF-1* demonstrated significant increases in cell death (compared to the same lines transduced the control siRNA) at 48 h post-transfection, which was not observed in si*NCF-1*-treated HeLa cells (Fig. 3*B*, middle panel). Cell death triggered by si*NCF-1* treatment was not observed in HeLa cells (Fig. 3*B*, right panel). Quantification of cell proliferative assay confirmed this result, showing that over 50% of both BxPC-3 and HSC-58 cells died within 2 to 3 days after transfection with si*NCF-1*, in contrast to the case with HeLa (Fig. 3*C*). These cytotoxic effects of si*NCF-1* transfection reflected apoptotic cell death, as demonstrated by the detection of the cleaved form of poly-ADP ribose polymerase (PARP; a marker of apoptosis) by immunoblotting (Fig. 3*D*).

We noted that NCF-1 is an essential component of NADPH oxidase, an enzyme that serves an important role in the production of ROS in neutrophils. Therefore, the cells with or without RNA interference by si*NCF-1* were incubated with a fluorescent ROS tracker to detect intracellular ROS production. Interestingly, the strong intracellular fluorescent signal corresponding to an accumulation of ROS localized at the growth front of tumor nests in BxPC-3 cells transfected with the control siRNA, indicating the production of high amounts of ROS during cell proliferation and/or cell motility in this cell line (Fig. 3*E*, upper panel). In contrast, the accumulation of ROS was not seen in si*NCF-1*-transfected BxPC-3 cells (Fig. 3*E*, upper panel). Similarly, HSC-58, which grows as individual cells (rather than forming tumor cell nests), displayed ROS accumulation in cultures transfected with the control siRNA, but ROS production was attenuated in si*NCF-1*-transfected HCS-58 cells. These results suggested that the decrease in ROS production (as seen in si*NCF-1*-transfected cells) is associated with cell death (Fig. 3*E*, lower panel).

### Shared accumulation of the ROS-regulatory molecules (including GSS, GSH, and GPX4) in NCF-1-expressing tumor cells

We next examined the effect, in tumor cells, of NCF-1 expression on the intracellular levels of regulators (glutathione synthetase (GSS) and glutathione peroxidase 4 (GPX4)) and a mediator (glutathione (GSH)) of ROS production. Notably, GSS, GSH, and GPX4 have been reported to play a critical role as determinants of cellular fate in response to ROS. Specifically, GSS catalyzes the intracellular synthesis of GSH. GPX4 activity prevents cellular injury (ferroptosis) that otherwise results from the accumulation of lipid ROS; this enzyme utilizes GSH for conversion of lipid hydroperoxides to lipid alcohols. As assessed by IHC of patient tumor tissues, the cytoplasmic expression of both GSS and GPX4 was detected on tumor cells of gastric adenocarcinoma and PDAC, as well as in lymph nodal metastatic sites of PDAC (Fig. 3*A* and *B*). In vitro analysis of BxPC-3 cells revealed that the accumulation of GSH was observed coincidentally in cells exhibiting strong accumulation of NCF-1; in contrast, the GSH signal appeared to be attenuated in cells in which NCF-1 was depleted as a result of RNA interference (Fig. 3*C*, upper panel). Similar phenomena were observed in HSC-58, in which cells exhibiting strong GSH accumulation corresponded to those that accumulated high levels of NCF-1 (Fig. 3*C*, lower panel).

**Figure 4.**
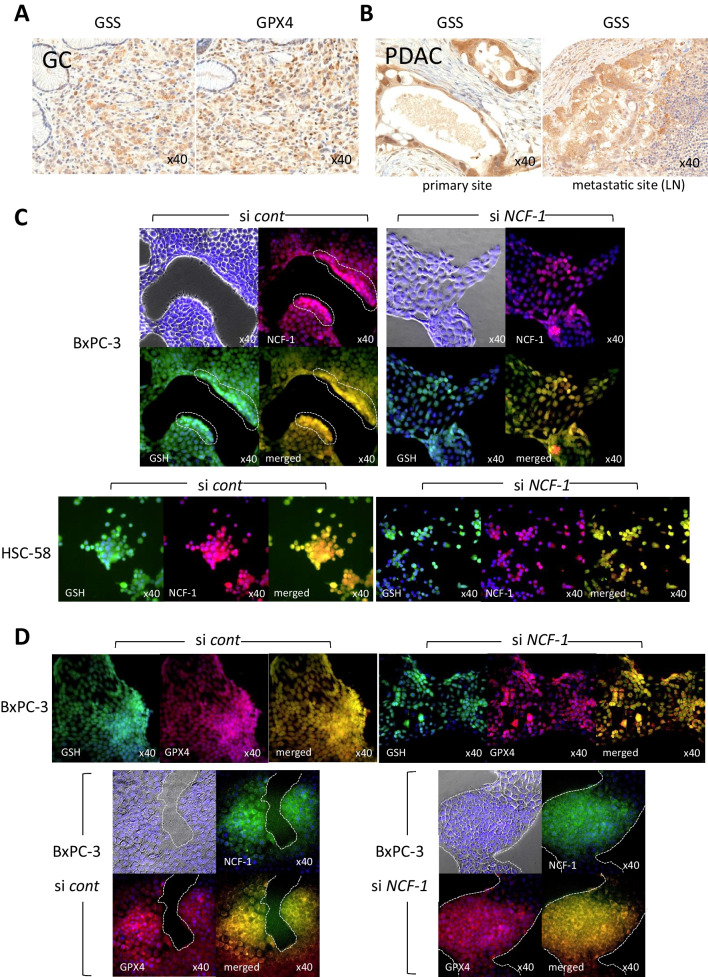
Expression of reactive oxygen species (ROS)–associated molecules (glutathione synthetase (GSS), glutathione peroxidase 4 (GPX4)) and accumulation of reduced glutathione (GSH) in patient tumor tissues and si*NCF-1*-transfected tumor cell lines. (*A*) Immunohistochemistry demonstrates prominent expression of GSS and GPX4 in patient gastric adenocarcinoma (gastric poorly differentiated adenocarcinoma; GC). (*B*) Expression of GSS in pancreatic adenocarcinoma tissue from a patient with pancreatic adenocarcinoma (PDAC) and metastatic PDAC to the lymph node (LN) (× 40). (*C*) Accumulation of GSH was coincident with strong NCF-1 expression in control siRNA- and si*NCF-1*-transfected tumor cells. BxPC-3 and HSC-58 cells were examined 96 and 48 h post-transfection (respectively) using Alexa488-labeled GSH-tracer and immunofluorescence with anti-NCF-1 antibody. The focused area of the cell nest is indicated by *hatched line*. (*D*) Accumulation of GSH was observed on the cells with strong expression of GPX4 (*upper panel*), and coexpression of NCF-1 and GPX4 (*lower panel*) in the same samples as were used in *C*. The boarder of the tumor cell nest is indicated by *hatched line*. All cellular images, × 40. Nuclei were counterstained with Hoechst 33258.

We next examined GPX4 expression in these tumor cells. This enzyme is a key factor protecting cells from ROS-induced cell death; in this context, GPX4 is an inhibitor of ferroptosis. We observed GPX4 accumulation in tumor cells that also accumulated GSH; notably, the level of GPX4 appeared to be affected by that of NCF-1, as indicated by a comparison between cells transfected with the control and *NCF-1* siRNAs (Fig. 3*D*, upper panel, GSH and GPX4; lower panel, NCF-1 and GPX4). Taken together, these results implied that NCF-1-producing tumor cells are resistant to ROS-mediated cytotoxic effects. These observations further suggested that the strong accumulation of ROS in these tumor cells might contribute to another biological aspect of ROS, namely, a role in the survival and growth of tumor cells (Fig. 4).

## Discussion

Reactive oxygen species have been reported to play a pivotal role in cellular fate, facilitating the choice between cell survival (growth) and cell death (Acharya *et al.* 2010; Trachootham *et al.* 2010). The biological role of ROS is reciprocal depending on cellular conditions, as shown by inverse effects of ROS when present at different intracellular concentrations. In tumor cells, elevated levels of ROS have been reported to cause alterations in the redox balance, leading to dysregulated redox signaling, a common hallmark of cancer progression and resistance to treatment with antitumor agents. Such increases in the production of ROS historically have been thought to be triggered by elevations in metabolic activity, cellular signaling, peroxisomal activity, mitochondrial dysfunction, oncogene activation, and oxidase activity in cancer cells (Kumari *et al.* 2018). Therefore, to identify key molecules that regulate the production and activation of intracellular ROS, it is important to understand live tumor biology. In the present study, we assessed the accumulation of NCF-1/p47phox, a subunit of an oxidase responsible for ROS generation in neutrophils (Groemping *et al.* 2003; Fang *et al.* 2015; Belambri *et al.* 2018). Our results demonstrated that NCF-1 is found not only in inflammatory cells but also in tumor cells of gastric and pancreatic origins, where the protein appears to play an essential role in the survival of these tumor cells. In vitro RNA interference assays revealed that depletion of NCF-1 induces cell death in both BxPC-3 and HSC-58 cells, indicating that NCF-1 is essential for the survival of such tumor cells. In contrast, the survival of a cervical adenocarcinoma cell line (HeLa) was not significantly affected by transfection with si*NCF-1*. Together, these results indicate that NCF-1 may not be essential for cell survival in all types of cancer, a distinction that may reflect different mechanisms of growth in tumors of distinct etiology. As mentioned above, ROS has a biphasic effect, such that different levels of ROS may exhibit cytotoxicity or growth stimulation in both non-neoplastic and tumor cells. In pancreatic and gastric cancers, NCF-1 appears to facilitate use of the ROS-production pathway for cancer cell survival and growth, an effect that may be mediated by the production of GSH by GSS (Njålsson R 2005; Nefedova *et al.* 2007; Zhu *et al.* 2023). Moreover, GPX4 may counteract cell death (ferroptosis) in proliferating tumor cells, a fate that otherwise would be triggered by the oxidative stress associated with excess ROS (Cao & Dixon 2016; Sekhar *et al.* 2022). In this context, the intracellular accumulation both of reduced GSH and GPX4 might promote cell survival and growth as these contributed to reduce toxicity of excess ROS in tumor cells. Thus, our results reveal a novel biological aspect of NCF-1 in cancer cells; we expect that further investigation of this phenomenon may contribute to elucidation of the detailed mechanism of ROS and redox regulation in pancreatic and gastric cancers.

## Supplementary Information

Below is the link to the electronic supplementary material.

Supplementary file1 (PPTX 108 KB)

## Data Availability

Correspondence and requests for materials should be addressed to Eisaku Kondo. Data generated and analyzed during the current study are available from the corresponding author upon reasonable request.

## References

[CR1] Belambri SA, Marzaioli V, Hurtado-Nedelec M, Pintard C, Liang S, Liu Y, Boussetta T, Gougerot-Pocidalo MA, Ye RD, Dang PM, El-Benna J (2022) Impaired p47phox phosphorylation in neutrophils from patients with p67phox-deficient chronic granulomatous disease. Blood 139(16):2512–2522 35108370 10.1182/blood.2021011134

[CR2] Belambri SA, Rolas L, Raad H, Hurtado-Nedelec M, Dang PM, El-Benna J (2018) NADPH oxidase activation in neutrophils: role of the phosphorylation of its subunits. Eur J Clin Invest 48(Suppl 2):e12951 29757466 10.1111/eci.12951

[CR3] Cao JY, Dixon SJ (2016) Mechanisms of ferroptosis. Cell Mol Life Sci 73(11–12):2195–2209 27048822 10.1007/s00018-016-2194-1 PMC4887533

[CR4] Charya A, Das I, Chandhok D, Saha T, Redox Regulation in Cancer (2010) A double-edged sword with therapeutic potential. Oxid Med Cell Longev 3:23–34 20716925 10.4161/oxim.3.1.10095 PMC2835886

[CR5] Fang X, Ying L, Lei S, Wei L, Li Z, Hongjing C, Jie Q, Yong C, Weichen W, Yejia H (2015) NADPH oxidase p47phox siRNA attenuates adventitial fibroblasts proliferation and migration in apoE(-/-) mouse. J Transl Med 28(13):38 10.1186/s12967-015-0407-2 PMC4312606 25628043

[CR6] Groemping Y, Lapouge K, Smerdon SJ, Rittinger K (2003) Molecular basis of phosphorylation-induced activation of the NADPH oxidase. Cell 113(3):343–55 12732142 10.1016/s0092-8674(03)00314-3

[CR7] Kumari S, Kumar Badana A, Murali Mohan G, Shailender G, Malla RR (2018) Reactive oxygen species: a key constituent in cancer survival. Biomark Insights 13:1177271918755391 29449774 10.1177/1177271918755391 PMC5808965

[CR8] Meijles DN, Fan LM, Howlin BJ, Li JM (2014) Molecular insights of p47phox phosphorylation dynamics in the regulation of NADPH oxidase activation and superoxide production. J Biol Chem 289(33):22759–22770 24970888 10.1074/jbc.M114.561159 PMC4132782

[CR9] Nagarkoti S, Dubey M, Awasthi D, Kumar V, Chandra T, Kumar S, Dikshit M (2018) S-glutathionylation of p47phox sustains superoxide generation in activated neutrophils. Biochim Biophys Acta Mol Cell Res 2:444–454 10.1016/j.bbamcr.2017.11.014 29195919

[CR10] Nefedova Y, Fishman M, Sherman S, Wang X, Beg AA, Gabrilovich DI (2007) Mechanism of all-trans retinoic acid effect on tumor-associated myeloid-derived suppressor cells. Cancer Res 67(22):11021–11028 18006848 10.1158/0008-5472.CAN-07-2593

[CR11] Njålsson R (2005) Glutathione synthetase deficiency. Cell Mol Life Sci 62(17):1938–1945 15990954 10.1007/s00018-005-5163-7 PMC11139123

[CR12] Sekhar KR, Hanna DN, Cyr S, Baechle JJ, Kuravi S, Balusu R, Rathmell K, Baregamian N (2022) Glutathione peroxidase 4 inhibition induces ferroptosis and mTOR pathway suppression in thyroid cancer. Sci Rep 12(1):19396 36371529 10.1038/s41598-022-23906-2 PMC9653479

[CR13] Tlili A, Pintard C, Hurtado-Nedelec M, Liu D, Marzaioli V, Thieblemont N, Dang PM, El-Benna J (2023) ROCK2 interacts with p22phox to phosphorylate p47phox and to control NADPH oxidase activation in human monocytes. Proc Natl Acad Sci USA 120(3):e2209184120 36626553 10.1073/pnas.2209184120 PMC9934299

[CR14] Trachootham D, Lu W, Ogasawara MA, Rivera-Del Valle N, Huang P (2010) Redox regulation of cell survival. Antioxid Redox Signal 10(8):1343–1374 10.1089/ars.2007.1957 PMC2932530 18522489

[CR15] Zhu H, Cheng Y, Wang X, Yang X, Liu M, Liu J, Liu S, Wang H, Zhang A, Li R, Ye C, Zhang J, Gao J, Fu X, Wu B (2023) Gss deficiency causes age-related fertility impairment via ROS-triggered ferroptosis in the testes of mice. Cell Death Dis 14(12):845 38114454 10.1038/s41419-023-06359-x PMC10730895

