# Supplementary figures and images for: NCF-1 plays a pivotal role in the survival of adenocarcinoma cells of pancreatic and gastric origins
Source: In Vitro Cell Dev Biol Anim. 2024 Dec 12;60(10):1151–9. doi: 10.1007/s11626-024-00994-0 (PMC11655584; doi:10.1007/s11626-024-00994-0)

## Slide 1
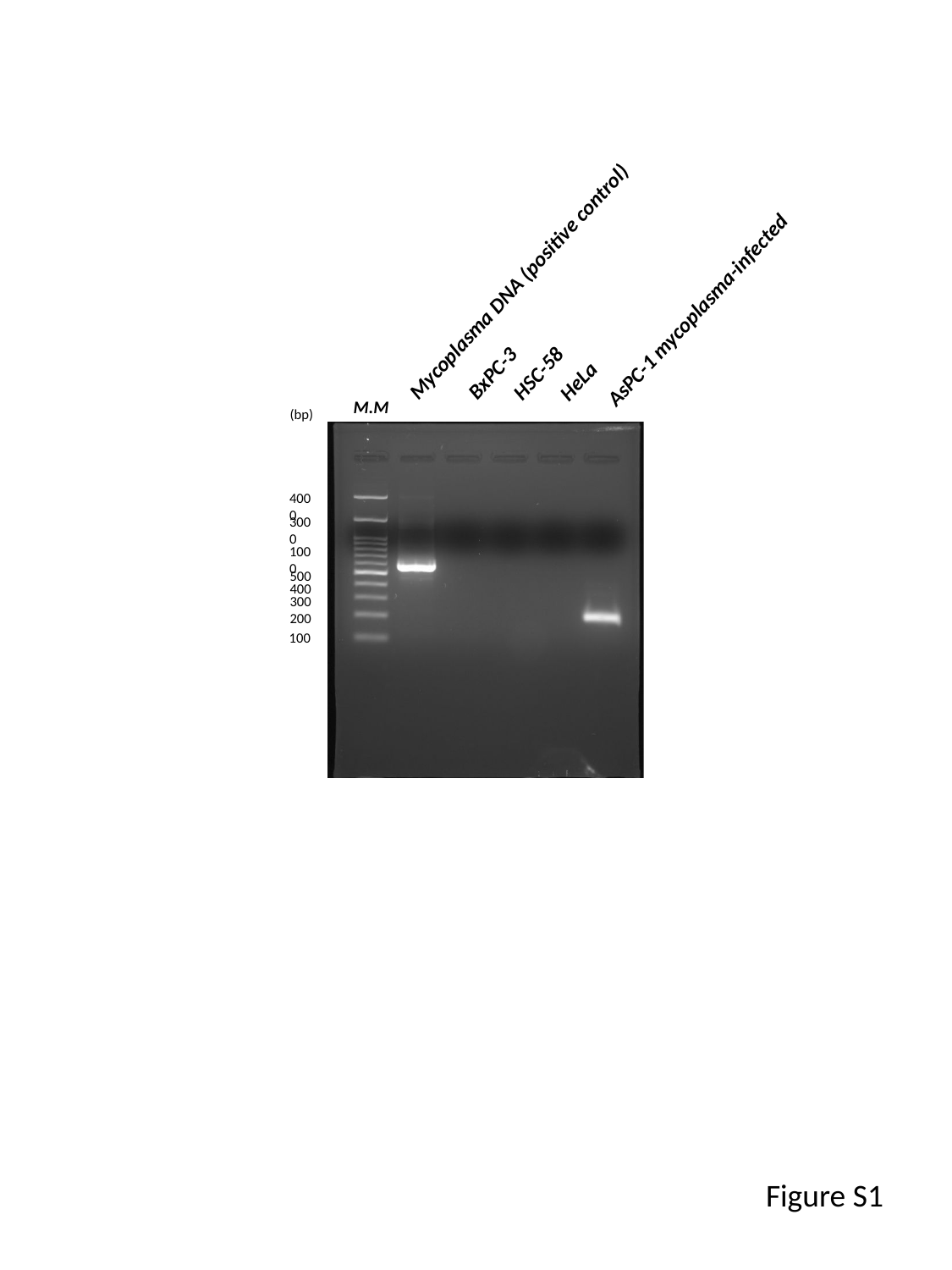

Mycoplasma DNA (positive control)
AsPC-1 mycoplasma-infected
BxPC-3
HSC-58
HeLa
M.M
(bp)
4000
3000
1000
500
400
300
200
100
Figure S1

Supplement: Supplementary file 1 — Supplementary file1 (PPTX 108 KB) [file 11626_2024_994_MOESM1_ESM.pptx]
